# Factors Associated with Bacterial Vaginosis among Women Who Have Sex with Women: A Systematic Review

**DOI:** 10.1371/journal.pone.0141905

**Published:** 2015-12-16

**Authors:** Dana S. Forcey, Lenka A. Vodstrcil, Jane S. Hocking, Christopher K. Fairley, Matthew Law, Ruth P. McNair, Catriona S. Bradshaw

**Affiliations:** 1 Melbourne Sexual Health Centre, Alfred Health, 580 Swanston Street, Carlton, 3053, Victoria, Australia; 2 Centre for Epidemiology and Biostatistics, Melbourne School of Population and Global Health, Level 5, 207 Bouverie Street, The University of Melbourne, Parkville, 3010, Victoria, Australia; 3 Central Clinical School, Monash University, The Alfred Centre, 55 Commercial Road, Melbourne, 3004, Victoria, Australia; 4 The Kirby Institute, The University of New South Wales, 101 West Street, Darlinghurst, 2010, New South Wales, Australia; 5 Department of General Practice, The University of Melbourne, Parkville, 3010, Victoria, Australia; University of Washington, UNITED STATES

## Abstract

**Background:**

Women who have sex with women (WSW) have a higher burden of bacterial vaginosis (BV) than heterosexual women; studies of risk factors specific to this population are limited. We summarised current knowledge regarding risk factors for BV among WSW by systematic review.

**Methods:**

This systematic review was conducted according to the PRISMA statement. PUBMED, EMBASE, Web of Science and The Cochrane Library were searched to 31^st^ December, 2014. Inclusion criteria: 1) WSW included in the study population; 2) accepted BV diagnostic method; 3) investigated or could extrapolate factors(s) associated with BV acquisition, persistence or transmission in WSW specifically by comparing BV positive to BV negative women. Search was limited to English-language publications.

**Results:**

A limited number of studies have investigated BV in WSW. Of 71 unique references, 18 full-text articles were assessed and 14 studies fulfilled inclusion criteria. BV was positively associated with higher numbers of female partners, both lifetime and in the three months prior to diagnosis, and confirmed BV in a female partner, but inconsistently associated with partners’ BV history or symptoms. BV was not associated with ethnicity, vaginal douching or hormonal contraception. The impact of specific sexual activities, male sexual contact, smoking and the menstrual cycle varied considerably between study populations.

**Conclusion:**

BV in WSW is associated with increased numbers of recent and past female partners and confirmed BV in a female partner. There are limited studies of BV in WSW populations, and research is needed to further elucidate risk factors for BV among WSW. However these data provide epidemiological evidence that BV risk in women is directly related to exposure to other female partners and a partner with BV, providing support for the concept that BV is likely to be transmitted between women.

**Systematic Review Registration Number:**

CRD42014009536 (PROSPERO)

## Background

Bacterial vaginosis (BV) is the most commonly identified cause of vaginitis in women of reproductive age.[1] BV has been linked to many sequelae including increased risk of pelvic inflammatory disease,[2] adverse obstetric outcomes,[3–7] HIV and STI acquisition [8] and HIV transmission.[9] BV recurrence after treatment is common and can negatively impact women’s emotional, social and sexual wellbeing.[10]

The majority of epidemiological studies of BV have been conducted in women who have sex with men (WSM) and have found consistent associations related to sexual risk exposure, including greater numbers of recent and lifetime male partners and inconsistent condom use.[11] Studies suggest that BV is highly prevalent in women who have sex with women (WSW), with estimates ranging from 25–50%,[12–16] In studies of WSW by our group and others, BV has been associated with several sexual activity risk factors, including increased number of female sexual partners[12,17,18], a female sexual partner with BV symptoms[14,19], and receptive oral sex.[19,20] Overall, the data on specific risk factors for BV in WSW are more limited than for WSM and no systematic review of risk factors of BV has been conducted.

We undertook a systematic review with the aim of establishing the risk factors associated with prevalent, incident, persistent or recurrent BV in WSW of any age by comparing women with BV to women without BV.

## Methods

### Protocol and registration

We used the PRISMA statement to guide this review (S1 Fig).[21] Methods for analysis, inclusion criteria and protocol were specified in advance and registered with PROSPERO, registration: CRD42014009536 (http://www.crd.york.ac.uk/PROSPERO/).

### Eligibility criteria

We searched for peer-reviewed, English-language studies published to 31^st^ December, 2014 that investigated BV in populations that included WSW. No time frame delimiters were specified and both cross-sectional and longitudinal studies were eligible. Identified conference abstracts were reviewed. Letters to the editor, focus group reports, case reports, case series, and review, editorial and discussion articles were excluded but their reference lists were examined.

We assessed all studies that investigated factors associated with prevalent, incident, persistent or recurrent BV in WSW of any age by comparing women with BV to women without BV. Studies were eligible for inclusion providing ≥1 variable was investigated in WSW alone; only results specific to WSW were included. Studies that exclusively enrolled WSM, sex workers or pregnant women were ineligible for inclusion. An established diagnostic method for BV, such as Amsel,[22] Nugent,[23] Spiegel[24]or Hay-Ison[25] criteria, was required for inclusion in the review.

### Information sources

Our search strategy was applied to the PUBMED, EMBASE, Web of Science and The Cochrane Library databases for studies published to 31^st^ December 2014.

### Search

The following search terms were applied: ((bacterial vaginosis) OR (BV) OR (vaginosis, bacterial) OR (bacterial infections and vaginitis) OR (vaginosis) OR (gardnerella)) AND ((WSW) OR (“women who have sex with women”) OR (lesbian) OR (“female homosexual”) OR (homosexuality, female)) AND Language = (English).

### Study selection

Abstracts of studies returned from the database searches were reviewed for eligibility and their citation lists searched for additional references. In cases of uncertainty regarding inclusion, the entire text was read. After removal of duplicates and ineligible results, full-text articles were reviewed.

### Data collection

A data extraction spread-sheet was created based on the Cochrane Consumers and Communication Review Group’s data extraction and assessment template and collated the data as described below.

### Data items

The following information was extracted: 1) study methods, design and aim, 2) population (sample size, numbers of WSW, country, recruitment site), 3) participant characteristics (age, WSW definition), 4) BV diagnostic method, 5) statistical analyses and 6) factors assessed for BV association.

### Analysis

Pooling of results for meta-analysis was not possible primarily due to the differences in how each risk factor was defined and/or presented and also because of the limited studies that contributed data for specific risk factors. We conducted a frequency analysis of factors associated with BV in WSW populations. The following were assessed for their association with BV: 1) Demographics (age, ethnicity), 2) Non-sexual behaviours (smoking, vaginal douching), 3) Hormonal influences (menstrual cycle, hormonal contraception), 4) Sexual behaviours with female and male sexual partners (FSP, MSP), 5) Diagnosed, self-report and history of BV in FSPs, and 6) BV-associated bacteria (BVAB), assessed by molecular methods.

### Risk of bias within individual studies

Assessment of bias was based on the STROBE guidelines for reporting observational epidemiological studies.[26] The parameters used were: 1) Selection bias: defined inclusion/ exclusion criteria, site/country of recruitment and recent female sexual contact; 2) Reporting bias: response rate for longitudinal studies; 3) Confounding: adjustment for confounding; and 4) for randomised controlled trials (RCT) and cohort studies: report of sample size calculations.

## Results

### Study selection

The literature search and assessment process is shown in S2 Fig. We identified 133 studies from initial searches of PUBMED (n = 44), The Cochrane Library (n = 7), EMBASE (n = 33) and Web of Science (n = 49). Additional records (n = 3) were identified from citation lists. Duplicate records (n = 65) were removed and 71 unique references were assessed, with 53 excluded on abstract review. The full texts of 18 articles were examined 14 of which were eligible for review. Four were excluded because they: did not provide the BV diagnostic method, reported factors associated with specific BV-associated bacteria but not with BV, did not stratify results by sexuality (WSW versus WSM) so it was not possible to extract results for WSW alone, or all women in the study had BV with no comparator group.

### Study characteristics

Of 14 eligible studies, there were 10 cross-sectional studies, three cohort studies and one RCT. (Table 1) All the included studies had ethics approval or detailed informed consent of participants. The majority of studies were from the USA, [13,15,18,27–30] the UK [14,16,31] and Australia.[12,19,32] Six studies were drawn from one research group [15,18,20,27,29,30] and two from another group.[12,19] Seven studies recruited from the community,[14,15,18,20,27,29,30] four from STI clinics or health services[13,16,28,31] and three recruited or used samples drawn from both.[12,19,32] Numbers of WSW participants ranged from 39 to 708. BV prevalence was investigated in 12 studies, two of which further investigated BV persistence rates; two studies investigated BV incidence. BV prevalence ranged from 12.4% to 51.6% in studies of WSW alone; one study that included symptomatic WSM and WSW had a BV prevalence of 56.2%.[32] In one study, 25.8% of BV persisted after treatment;[29] another investigated BV recurrence after a behavioural intervention, with rates of 21.1% in the control and 27.9% in the intervention groups.[30] Two longitudinal cohorts reported BV incident rates of 9.8 per 100 woman-years,[19] and another cohort diagnosed 40 BV episodes in 199 women, with a rate of 23 per 100 woman-years.[15] Results for factors assessed for association with BV are shown in Table 2, Table 3.

**Table 1:** Characteristics of studies included for review. Note: AS–Amsel Score, NS–Nugent Score

Study	Country	Study type	Study population (no. of individuals, ages if reported)	Setting	Number BV positive/ Total (%)	Diagnostic method(s)
Berger *et al*,1995 [13]	USA	Cross-sectional	103 WSW, including 21 monogamous couples	Gynaecology practice, community clinic	Prevalence 29 / 101 (28.7) – 2 ungradeable	AS
McCaffrey *et al*, 1999 [16]	UK	Cross-sectional	91 women	Specialist lesbian genitourinary clinic	Prevalence 47 / 91 (51.6)	Hay-Ison criteria
^a^Marrazzo *et al*,2002 [15]	USA	Cross-sectional	326 WSW, including 58 monogamous couples, age ≥16 years	Community recruitment	Prevalence 81 / 326 (25)	NS
Bailey *et al*, 2004 [31]	UK	Cross-sectional	708 WSW, age 16–53 years	New patients at a lesbian/ bisexual sexual health clinic	Prevalence 222 / 708 (31.4)	AS
Evans *et al*, 2007[14]	UK	Cross-sectional	171 WSW, 189 heterosexual women, age 16–50 years	Community recruitment	Prevalence (WSW) 43 / 167 (25.7) – 4 ungradeablePrevalence (WSM) 27 / 187 (14.4) – 2 ungradeable	Hay-Ison criteria
^bb^Marrazzo *et al*, 2008 [29]	USA	Observational cohort	335 WSW, age 16–30 years	Community recruitment	Prevalence 96 / 335 (28.7)Persistence 31 / 120 (25.8)	AS + NS
^aa^Marrazzo *et al*, 2009 [27]	USA	Cross-sectional	237 WSW, age ≥16 years	Community recruitment	Prevalence 14 / 237 (5.9)	NS + PCR
^b^Marrazzo *et al*, 2010 [18]	USA	Prospective cohort	335 WSW, age 16–35 years	Community recruitment	Prevalence 96 / 335 (28.7)	AS + NS + PCR
^b^Marrazzo *et al*, 2010 [20]	USA	Cross-sectional	335 WSW, age 16–35 years	Community recruitment	Incidence 40 / 199 (20.1)	AS
^bb^Marrazzo *et al*, 2011 [30]	USA	Randomised control trial	89 WSW, age 16–30 years	Community recruitment	Persistence 12 / 43 (27.9) intervention arm, Persistence 8 / 38 (21.1) control arm	AS + NS
^c^Fethers *et al*, 2012 [32]	Australia	Cross-sectional	193 women, age 17–21 years^c^	University recruitment	Prevalence 24 / 193 (12.4) ^c^	NS
			146 symptomatic women^c^	Sexual health clinic	Prevalence 82 / 146 (56.2) ^c^	
Muzny *et al*, 2013 [28]	USA	Cross-sectional	196 WSW, age ≥18 years	STD Clinic	Prevalence 93 / 196 (47.4)	AS + NS
Bradshaw *et al*, 2014 [12]	Australia	Cross-sectional	458 WSW, age 18–55 years	Community recruitment, STI clinic, GP Clinic	Prevalence 125 / 458 (27)	NS
Vodstrcil *et al*, 2014 [19]	Australia	Longitudinal cohort	298 WSW, age 17–55 years	Community recruitment, STI clinic, GP clinic	Incidence 51/298 (10 per 100PY)	NS

^aa^Sub-population (of study^a^); different variables investigated)

^b^Same study population (^bb^Sub-population; different outcomes measured: prevalent, incident and persistent BV)

^c^Includes both women who have sex with women (WSW), women who have sex with men (WSM) and women who have sex with women and men (WSWM)

**Table 2:** Variables investigated for association with BV in studies included for review. Blank where the variable not investigated or variable not stratified by WSW alone. Odds ratios/ Risk Ratio/ Hazard Ratio/ Proportions/ Kappa score for correlation of vaginal flora (95% confidence intervals) are displayed (**Bold** where significant) for factors associated with BV. Variables positively/ negatively associated with BV are from multivariate analyses unless otherwise indicated (P reported proportions only, ^U^ univariate analysis only). ^a^Same study population (^aa^Sub-population; different variables investigated), ^b^Same study population (^bb^Sub-population; different outcomes measured: prevalent, incident and persistent BV). */**/*** See ‘Notes’ column.–Data not shown; variable not associated with BV. FSP (female sexual partner), MSP (male sexual partner), m (months). BV (bacterial vaginosis), BVAB (bacterial vaginosis-associated bacteria)

	Berger *et al*, 1995 *[13]*	McCaffrey *et al*, 1999 [16]	^a^Marrazzo *et al*, 2002 [15]	Bailey *et al*, 2004 [31]	Evans *et al*, 2007[14]	^b^Marrazzo *et al*, 2008 [29]*	^aa^Marrazzo *et al*, 2009 [27]	^b^Marrazzo *et al*, 2010 [18]	^b^Marrazzo *et al*, 2010[20]	^bb^Marrazzo *et al*, 2011 [30]	Fethers *et al*, 2012 [32]	Muzny *et al*, 2013 [28]	Bradshaw *et al*, 2014 [12]	Vodstrcil *et al*, 2014 [19]
**Age**			31/81	0.97 (0.86,1.12)		1.0 (0.5,1.8)^U^		1.2 (0.85,1.69)^U^				0.86 (0.49,1.51)	0.97 (0.56,1.66)	1.22(0.65,2.31)
**Ethnicity**		3/6	1.4 (0.7,3.0)	2.48 (1.04,5.92)*		1.6 (0.0,4.2)^U^		1.71 (0.90,3.26)	1.0 (0.14,7.34) ^U^		*	*	-*	0.85(0.41,1.80)*
**Smoking**		21/36		**1.43 (1.01,2.03)**				–^U^	0.89 (0.47,1.68) ^U^			0.63 (0.35,1.12)	2.74 (1.49,5.04)	2.09(1.20,3.64)^U^**
**Douching**			1.5 (0.9,2.5)					1.53 (0.90,2.58)^U^	5/96			1.68 (0.81,3.49)	-	1.60(0.35,7.36)
**Hormonal contraception**					*			0.90 (0.48,1.67)^U^	9/96			0.30 (0.06,1.50)	**-**	-
**Menstrual cycle**								1.29 (0.92,1.82)	0.16 (0.07,0.36)*				**-**	0.85 (0.48,1.51)
**Sexual behaviours with FSP**														
**Receptive digital-vaginal sex**		47/89	81/81	0.77 (0.46,1.30)					0.74 (0.35,1.58) ^U^			0.96 (0.51,1.79)		2.27(0.97,5.31)
**Protected digital-vaginal sex**										1.12 (0.62,2.01)				
**Receptive digital-anal sex**		28/51	1.1 (0.6,2.0)	1.08 (0.60,1.93)		0.6 (0.00,1.4) *** ^U^								
**Receptive oral-vaginal sex**		46/90	80/81	0.86 (0.58,1.26)		1.1 (0.6,2.2) ** ^U^		1.05 (0.69,1.62)*	**1.02 (1.00,1.04)** ^U^**				**-**	3.52(1.41,8.79)***
**Receptive oral-anal sex**		17/33	**2.4 (1.3,4.4)**	1.06 (0.60,1.87)		0.6 (0.00,1.4) *** ^U^		1.36 (0.94,1.95)*	0.75 (0.29,1.92) ^U^*			1.95 (0.96,3.92)	**-**	
**Vaginal sex toy use**		26/47		0.86 (0.55,1.35)									**-**	
**Shared vaginal sex toy use**		17/34	2.7 (1.2,6.1)*					1.53 (1.09,2.28)	1.15 (0.51,2.63)^U^			1.09 (0.58,2.03)	**-**	1.97(1.0, 3.61) ^U^
**Increased # lifetime FSPs**		41/86 (>2 FSPs)		**1.60 (1.05,2.44)** (≥11 FSPs)								**0.08 (0.01,0.74)** (>1 FSP)	**1.92 (1.19,3.09)** (>4 FSPs)	
**Increased # of recent FSPs**		**21/38** (>1 FSP, 12m)		1.55 (1.04, 2.31)^U **^ (≥3 FSP, 1m)				1.58 (1.09,2.28) (>1 FSP, 3m)					1.6 (0.96,2.67) (>1 FSP, 12m)	2.51 (1.30,4.82)(New partner,3m)****
**BV history/ symptoms in FSP (self-report)**						0,9 (0.2,2.0) ^U^		2.55 (1.85,3.49)	2.96 (0.38,23.2)				**1.84 (1.23,3.02)**	3.99(1.39,11.45)
**BV diagnosed in FSP during study**	**19.7 (2.1,588.0) K = 0.62 (0.46,0.78)**		32/35		**K = 0.63 (0.46,0.81)**			11.4 (2.9,44.3) ^U^					**K = 0.47**	**K = 0.24(0.06,0.42)**
**Increased lifetime # MSPs**		31/59 (≥3 MSP)	1.1 (0.5,2.5) (≥7 MSP)	1.12 (0.70,1.79) (≥ 6 MSP)								1.63 (0.70,3.76) (>1 MSP)	**-**	
**Increased # recent MSPs/ New MSPs**				1.76 (0.58,5.32)(≥2 MSP, 12m)				0.68 (0.30,1.53) (>1 MSP, 3m)						2.51 (1.30,4.82) (New partner,3m)****
**Vaginal bacterial species**			*G*.*vaginalis*, *U*.*urealyticum*, Gram+ cocci, *Prevotella* spp, anerobic black-pigmented anaerobic Gram–rods, coagulase–Staphylococci associated with BV (P)			**BVAB3 2.6 (1.4,5.45), *P*.*lacrimalis* 2.8 (1.2, 13.3)**	*L*.*gasseri* in vagina/ rectum: 4.2 (1.4,13.4)^U^		***L*.*crispatus* 0.18(0.08,0.4)^U^, BVAB1 6.3 (1.4,28.1) ^U^, BVAB2 18.2(6.4,51.8)^U^*P*.*lacrimalis* 2.7 (1.1,6.4) ^U^, *G*.*vaginalis* 3.9 (1.5,10.4) ^U^, *Atopobium* 4.2 (1.9,9.0) ^U^, *Leptotrichia* 9.3 (3.6,24.4) ^U^, *Megasphera t*ype I 11.5 (5.0,26.6)^U^**		***Megasphera* type I in BV+ women associated with WSW, 4.4(1.2,16.4)**			
Notes			*Not always cleaning vaginal sex toy between use on subject and on a partner	*Asian ethnicity, **Not included in multivariable model	*Only WSM asked	*BV persistence, **With FSP or MSP, ***Includes all anal sex behaviours		*With MSPs and FSPs, **Highly correlated with sex toy use	*≤14 days since menses (incident BV), **Dose-response, per act		*Enquired about country of birth; not associated with prevalent BV	*Investigated African-American women only	*Enquired about country of birth; not associated with prevalent BV	*Enquired about country of birth; not associated with incident BV** Correlated with FSP non co-enrolment***From FSP or MSP****Combined FSP, MSP (mainly reflects FSP)

**Table 3:** Summary of associations with prevalent or incident/recurrent/persistent BV.

	Type of study design			
	Prevalence		Incidence / Recurrence / Persistence	
Variable	Total number of studies investigating an association	Number of studies reporting a significant association	Total number of studies investigating an association	Number of studies reporting a significant association
Age	5	0	3	0
Ethnicity	4	1	2	0
Smoking	5	2	3	1
Vaginal douching	5	0	3	0
Hormonal contraception	3	0	3	0
Menstrual cycle	2	0	2	1
Receptive digital-vaginal sex with FSP	5	0	2	0
Protected digital-vaginal sex with FSP	0	0	1	0
Receptive digital-anal sex with FSP	5	0	0	0
Receptive oral-vaginal sex with FSP	5	0	3	2
Receptive oral-anal sex with FSP	5	1	1	0
Vaginal sex toy use	3	0	0	0
Shared vaginal sex toy use	4	2	2	1
Increased number of lifetime FSPs	6	5	0	0
Increased number of recent FSPs/ New FSP	3	1	2	1^e^
BV history/symptoms in FSP (self-report)	2	2	3	1
BV diagnosed in FSP during study	4	4^d^	1	1
Increased number of lifetime MSPs	5	1	0	0
Increased number of recent MSPs/ New MSPs	2	0	1	1^e^

^d^Defined in recent sexual partner(s) in cross-sectional studies (either within last 12 months, or last 3 months), or a new sexual partner in longitudinal cohort studies.

^e^Significant on univariate analysis; significant on multivariate analysis when new FSP/MSP was combined with new MSP/FSP in a broader “new partner” category but for the majority of women this represented a new FSP. FSP (female sexual partner), MSP (male sexual partner)

### Demographics

None of the eight studies that investigated age found any association with prevalent [12,15,19,20,28,29,31] or incident [19] BV in WSW. Participant ethnicity was investigated in six studies,[15,16,18,20,29,31] one investigated BV risk factors in African-American WSW alone.[28] Three studies reported country of birth, which was not associated with prevalent BV.[12,19,32] One study found a positive association between BV and Asian ethnicity;[31] no others found associations between ethnicity and BV.

### Non-sexual behaviours

The association between smoking and BV in WSW was reported in eight studies.[12,16,18–20,28,29,31] Three demonstrated a positive association with BV.[12,19,31] Smoking was associated with incident BV in WSW on univariate analysis in one study, but was not included in the multivariate analysis due to correlation with other variables.[19] There was no association between smoking and incident or prevalent BV in three studies from one group,[18,20,29] or two additional studies.[16,28] Two studies did not stratify smoking according to sexual behaviour (WSW versus WSM) so the association could not be investigated for WSW.[14,32]

None of the eight studies that examined associations between vaginal douching and BV in WSW[12,15,18–20,28,29,31] reported an association with BV, although douching was positively associated with ‘abnormal’ vaginal flora (NS 4–10) in one.[15] Two studies did not stratify douching according to sexual behaviour.[14,32]

### Hormonal influences

Hormonal contraceptive use was relatively uncommon in WSW, ranging from 4–13% in the six studies that investigated its association with BV,[12,18–20,28,29] and it was not found to be associated with BV. In one study, 41% of participants used hormonal contraception but this was not stratified by sexual behaviour[32] and in another study contraceptive use was investigated only in heterosexual women.[14]

Results from four studies that investigated associations between BV and stage in the menstrual cycle were mixed. Two studies found no association;[12,19] another group found that ≤14 days since menses onset was positively associated with incident BV,[20] but not with prevalent BV.[18]

### Sexual activities

Sexual risk factors for BV in WSW were evaluated in 11 studies.[12,14–16,18–20,28–31] Most significantly, five of the six studies that investigated numbers of recent or lifetime FSPs demonstrated a positive association between prevalent BV and increased numbers of lifetime FSPs.[12,14–16,28,31] Higher numbers of recent female partners, including >1 FSP in the preceding 3 months, was also associated with prevalent[18] and incident[19] BV, though report of a new FSP within 12 months was not associated with an increased risk in one study.[12] Conversely, one study found that BV was less common in women who reported >1 lifetime FSP.[20] Increasing frequency of sex with any partner (though largely reflecting sex with a FSP) was significantly associated with BV[19] and a direct dose-dependent relationship was seen for BV and episodes of receptive oral-vaginal sex in one study.[20] No specific sexual practices with FSPs were consistently associated with BV. Receptive oral-vaginal sex was not associated with prevalent BV in six studies [12,15,16,18,29,31] but was associated with incident BV in two studies, [19,20] one of which found an association with increasing frequency of oral sex.[20] Two incidence studies investigated anal sex activities, one found no association between BV and oral-anal sex,[20] the other found no association with BV and any anal sex activities and incident BV.[29] Receptive oral-anal sex was positively associated with prevalent BV in one study[15] but not in four others.[12,16,28,31] Sharing vaginal sex toys was investigated in six studies and was positively associated with prevalent BV in two studies[15,18] and incident BV in one study,[19] but this was not the case in other studies.[16,28,29] There was no association between BV and receptive digital-vaginal sex[12,15,16,19,20,28,31] or receptive digital-anal sex.[12,15,16,18,31] A behavioural trial successfully increased glove use for digital sex but did not alter BV persistence.[30]

Sexual contact with males did not increase the odds of prevalent BV or risk of BV acquisition in WSW. Five of seven studies that investigated this factor found no association between BV and number of lifetime[15,16,31] or recent MSPs,[20,31] and one study of African-American WSW found that BV prevalence was lower in women reporting one lifetime MSP than women reporting >1 lifetime MSPs.[28]

### BV in female sexual partners

Five studies examined the association between BV and having a female partner concurrently diagnosed with BV by Amsel criteria[13] or Nugent score.[12,14,15,19] All showed positive associations with BV prevalence and incidence. BV was less reliably associated with self-report of a female partner’s BV than with a partner’s BV diagnosed using an established method. Whilst history of a partner with BV was positively associated with prevalent BV[18] and self-report of a partner with BV symptoms was positively associated with BV prevalence [12] and incidence[19], Marrazzo and colleagues found no association between self-report of a partner with BV and BV prevalence[20] or persistence,[29] once adjusting for other factors.

### BV-associated bacteria

Five studies examined the association between BV and vaginal bacterial species by molecular methods.[15,20,27,29,32] Bacteria positively associated with BV in WSW included *G*.*vaginalis*,[15,32] *A*. *vaginae*,[20,32] *Megasphera* type I,[32] *U*.*urealyticum*,[15] *Prevotella* spp.[15], coagulase-negative staphylococci,[15] anaerobic gram-positive cocci,[15] and gram-negative rods.[15] One study found BVAB3 and *P*.*lacrimalis* were associated with persistent BV.[29] Women with vaginal or rectal colonisation with *L*.*gasseri* were more likely to have prevalent BV[27] but women with *L*.*crispatus* at baseline were less likely to develop BV over the next 12 months.[20] Fethers *et al* [32] included WSM, but found detection of *Megasphera I* was associated with BV in WSW.

### Assessment of bias

Table 4 displays the assessment tools. Table 5 displays the results for reviewed papers. Inclusion criteria were described in 12 studies,[12–15,18–20,27–30,32] and six described exclusion criteria.[12–14,19,30,32] Recent female sexual contact was an inclusion criterion in 10 studies.[12,15,18–20,27–30,32] Others recruited women with any lifetime female sexual contact,[13] self-identifying WSW[14] or from a lesbian/ bisexual health clinic.[16,31]

**Table 4:** Method for assessment of internal and statistical validity of studies included for review. Some criteria not applicable depending on study design, these boxes left blank in ***Table 5***.

Bias		Criteria	Symbol in Table 5
**Selection bias**	Inclusion and exclusion criteria	Described	✓
		Not described	X
	Site of recruitment	Described	✓
		Not described	X
	FSP contact for WSW definition	Sexual contact with FSP within a specific time frame required for inclusion/ characterisation as WSW	✓
		No timeframe specified OR lifetime FSP OR population drawn from attendees at a WSW sexual health clinic (i.e. Assumed FSP contact) OR self-identifying WSW without specified criteria	X
**Reporting bias**	Response rate reported (Longitudinal studies)	Reported	✓
		Not reported	X
**Confounding**	Adjustment for confounding	Univariate analysis (not adjusted)	X
		Multivariate analysis (adjusted)	✓
**Sample size calculations**	Sample size calculations (RCTs, cohort studies)	Described	✓
		Not described	X

**Table 5:** Assessment of internal and statistical validity of studies included for review. Blank where criteria not required due to study design.

Study	Selection bias				Reporting bias	Confounding	Sample size
	Inclusion criteria	Exclusion criteria	Site of recruitment	Recent FSP for WSW definition	Response rate reported	Adjustment for confounding	Sample size statement
Berger *et al*, 1995 *[13]*	✓	✓	✓	X (Lifetime contact with FSP)		X	
McCaffrey *et al*, 1999 [16]	X	X	✓	X (Attending lesbian sexual health clinic)		X	
^a^Marrazzo *et al*, 2002 [15]	✓	X	✓	✓		✓	
Bailey *et al*, 2004 [31]	X	X	✓	X (Attending lesbian/ bisexual health clinic)		✓	
Evans *et al*, 2007[14]	✓	✓	✓	X (Self-identifying)		✓	
^b^Marrazzo *et al*, 2008 [29]	✓	X	✓	✓		Adjusted for one key variable; small numbers preclude multivariate analysis	
^aa^Marrazzo *et al*, 2009 [27]	✓	X	✓	✓		Univariate analyses	
^b^Marrazzo *et al*, 2010 [18]	✓	X	✓	✓	✓	✓	X
^b^Marrazzo *et al*, 2010 [20]	✓	X	✓	✓		✓	
^bb^Marrazzo *et al*, 2011 [30]	✓	✓	✓	✓	✓	✓	✓
^c^Fethers *et al*, 2012 [32]	✓	✓	✓	✓		✓	
Muzny *et al*, 2013 [28]	✓	X	✓	✓		✓	
Bradshaw *et al*, 2014 [12]	✓	✓	✓	✓		✓	
Vodstrcil *et al*, 2014 [19]	✓	✓	✓	✓	✓	✓	✓

^aa^Sub-population (of study^a^); different variables investigated)

^b^Same study population (^bb^Sub-population; different outcomes measured: prevalent, incident and persistent BV)

^c^Includes both women who have sex with women (WSW), women who have sex with men (WSM) and women who have sex with women and men (WSWM)

Confounding was adjusted for in 10 studies.[12,14,15,18–20,28,30–32] One study investigating BV persistence adjusted for treatment non-adherence but small participant numbers precluded multivariate analyses.[29] Three studies did not analyse confounders.[13,16,27] A longitudinal cohort study and RCT reported sample size calculations;[19,30] one prospective cohort study did not report sample size calculations.[18]

## Discussion

To our knowledge, this is the first systematic review of risk factors associated with BV in women who have sex with women. In this population, we found that prevalent and incident BV were associated with increased number of lifetime and recent FSPs and having a female partner with confirmed BV. However, unlike studies in WSM, no association between BV and ethnicity, vaginal douching or hormonal contraception was found. The association between BV and exposure to increased numbers of female partners and a female partner with BV together with a high concordance of BV between female sexual partners in published studies supports the concept that BV is likely sexually transmitted between women. These findings further support a previous meta-analysis that showed that having a FSP is associated with a 2-fold increased risk of BV.[11]

Our review indicates that among women with female partners, the presence of BV in one’s partner was consistently associated with BV in the index. This association was most robust when the partner’s BV was diagnosed using an established method, rather than reliance on self-report of a partner’s BV history or symptoms, which may be inaccurate as BV knowledge is often low, even in high-risk groups.[33] Increased number of recent and lifetime FSPs were positively associated with prevalent and incident BV in WSW but among WSW who also had sex with men, there was no association between increased number of recent and lifetime male partners and BV. If BV is transmitted between women, the association between BV and increased numbers of FSPs may reflect the high probability of encountering BV in a new partnership drawn from a population with relatively high overall prevalence estimates (25–50%). By comparison, one cohort study found a greatly reduced risk of BV acquisition for women who were both BV negative at enrolment and remained in that relationship over two years.[19] Importantly, no specific sexual activities were consistently associated with BV in WSW in this review, however some studies demonstrate an increased risk of BV acquisition with increasing frequency of sexual contact. This may be due to the ubiquitous nature of many sexual practices and the rarity with which sexual behaviours occur in isolation.

Many BVAB identified in WSW are also associated with BV in WSM,[32,34] however some differences may exist in the composition of the vaginal bacterial communities between WSW and WSM. One study found that *Megasphera I* detection was particularly sensitive for the diagnosis of BV (by Nugent criteria) in WSW.[32] Others have found that BV-positive WSW have higher levels of *Prevotella* spp. and lower vaginal bacterial diversity compared to WSM.[35] Overall there are comparatively fewer studies examining the vaginal microbiota in WSW compared to studies in WSM.

BV was not associated with age, ethnicity, vaginal douching or hormonal contraceptive use in WSW. Studies of WSM demonstrate reduced risk of prevalent, incident and recurrent BV in women using hormonal contraception,[36] and increased risk of BV in WSM that douche,[37,38] but the low rates of these practises in WSW enrolled in the included studies may have limited the power of included studies to detect any association with BV. The observation that BV was not associated with ethnicity in WSW but was in WSM may indicate that it is not an independent risk factor, but confounded by unmeasured sexual risk.

BV was inconsistently associated with smoking despite it being a risk factor in WSM.[39] Rates of smoking in WSW are generally high,[40] and may be correlated with other factors,[19] which could have limited our ability to examine the effect of smoking as an independent risk factor for BV. Similarly, the association between stage in the menstrual cycle and BV in WSW was also inconclusive. Studies of WSM suggest that BV is more common in the first 7 days of the menstrual cycle.[41] However, few studies investigated this variable in WSW, limiting our ability to assess associations.

This review has a number of limitations to be considered. We may have omitted relevant results by limiting our search to English language publications and by limiting inclusion to published, peer-reviewed papers to control for quality, we may have omitted otherwise relevant conference abstracts and other non-peer reviewed literature. All studies were undertaken in high income countries: the USA, UK and Australia and most studies investigated predominantly Caucasian populations, [14–16,18,20,27,29–31] which may limit the generalizability of our findings to WSW in the broader community and other countries. Despite the high prevalence estimates of BV in WSW, few studies have investigated BV in this population. Although we endeavoured to include all eligible published literature, several research groups dominated publications, and our findings may therefore have placed undue importance on the findings of these groups.

A strength of this review is the broad assessment of studies’ potential biases. In particular, we considered studies’ definition of ‘WSW’ in assessing potential selection bias. Despite the variety of ways to define sexuality, sexual activities with partners of a specific sex may provide the most relevant definition for studies investigating sexual risk factors for disease. Studies that recruited self-identifying WSW or women attending lesbian/bisexual health clinics may involve women who identify as lesbian or bisexual but have no female sexual contact.[40] It is important to consider that WSW with and without male partners may have different epidemiological risks, not just due to partner’s sex but to differences in sexual behaviours, hormonal contraceptive use, lubricants and condom use.

## Conclusion

Our systematic review of BV in WSW found evidence that BV among this population is associated with increased number of female partners and having a partner having confirmed BV. These epidemiological data have important implications for our understanding of BV pathogenesis, providing indirect evidence of BV transmission between women and their female partners. These data inform our current clinical management approaches and highlight the need to consider partner screening and treatment in BV-positive women and female partner treatment trials.

## Supporting Information

S1 Fig PRISMA Checklist: PRISMA checklist for factors included for this systematic review. *From*: Moher D, Liberati A, Tetzlaff J, Altman DG, The PRISMA Group (2009). Preferred Reporting Items for Systematic Reviews and Meta-Analyses: The PRISMA Statement. PLoS Med 6(6): e1000097. doi:10.1371/journal.pmed1000097
(DOC)

S2 Fig PRISMA flow diagram. PRISMA flow diagram for selection of studies for the systematic review of risk factors for BV among WSW. From Moher D, Liberati A, Tetzlaff J, Altman DG, The PRISMA Group (2009). Preferred Reporting Items for Systematic Reviews and Meta-Analyses: The PRISMA Statement. PLoS Med 6(6): e1000097. doi:10.1371/journal.pmed1000097
(TIFF)

## Abbreviations

BV: Bacterial vaginosis
WSM: women who have sex with men
WSW: women who have sex with women
FSP: female sexual partner
MSP: male sexual partner
BVAB: Bacterial vaginosis-associated bacteria
RCT: randomised control trial
