# Supplementary figures and images for: Factors Associated with Bacterial Vaginosis among Women Who Have Sex with Women: A Systematic Review
Source: PLoS One. 2015 Dec 16;10(12):e0141905. doi: 10.1371/journal.pone.0141905 (PMC4682944; doi:10.1371/journal.pone.0141905)

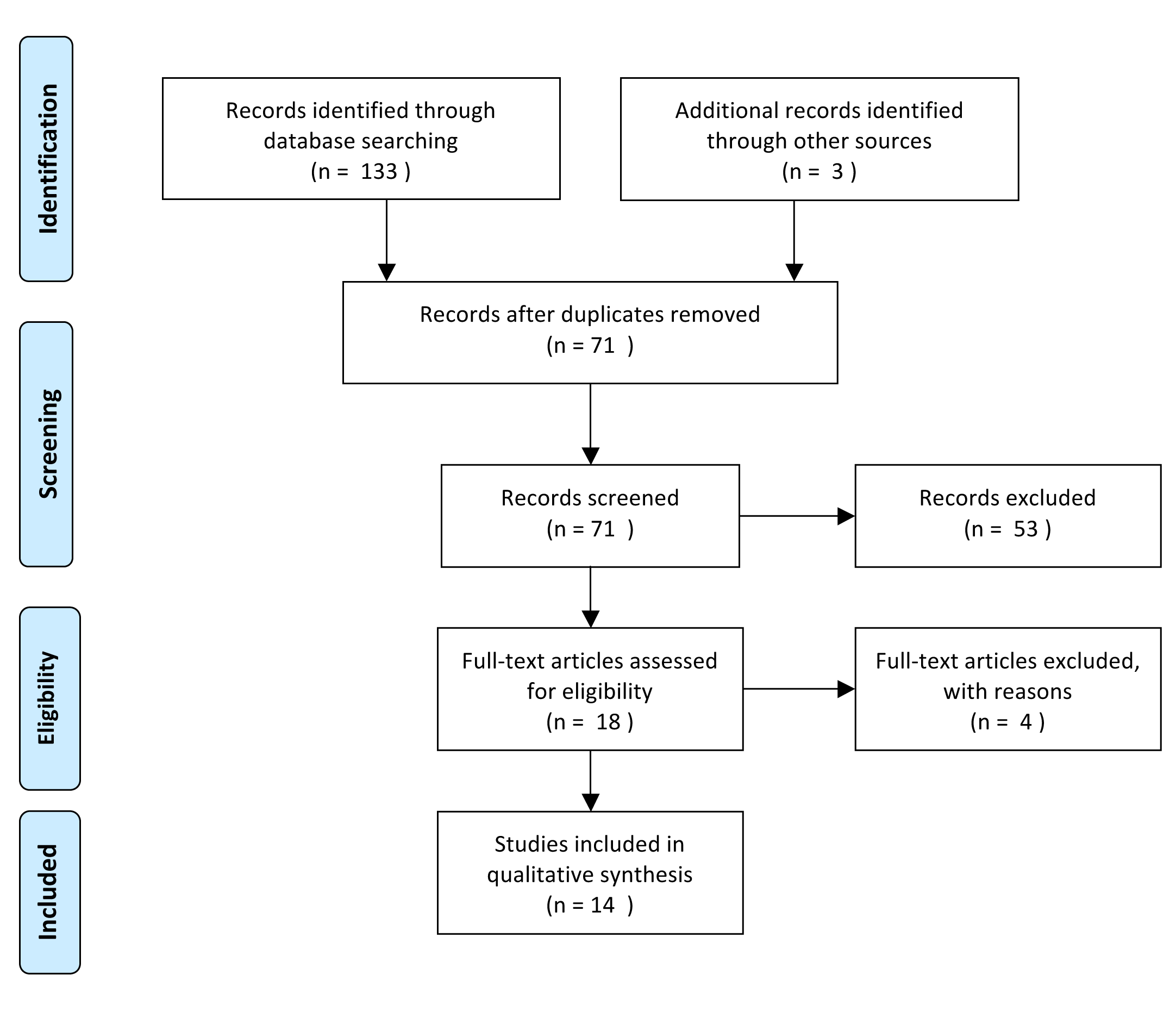

Supplement: S2 Fig — PRISMA flow diagram for selection of studies for the systematic review of risk factors for BV among WSW. From Moher D, Liberati A, Tetzlaff J, Altman DG, The PRISMA Group (2009). Preferred Reporting Items for Systematic Reviews and Meta-Analyses: The PRISMA Statement. PLoS Med 6(6): e1000097. doi:10.1371/journal.pmed1000097 (TIFF) [file pone.0141905.s002.tiff]
